# Reproductive factors and risk of hormone receptor positive and negative breast cancer: a cohort study

**DOI:** 10.1186/1471-2407-13-584

**Published:** 2013-12-09

**Authors:** Rebecca Ritte, Kaja Tikk, Annekatrin Lukanova, Anne Tjønneland, Anja Olsen, Kim Overvad, Laure Dossus, Agnès Fournier, Françoise Clavel-Chapelon, Verena Grote, Heiner Boeing, Krasimira Aleksandrova, Antonia Trichopoulou, Pagona Lagiou, Dimitrios Trichopoulos, Domenico Palli, Franco Berrino, Amalia Mattiello, Rosario Tumino, Carlotta Sacerdote, José Ramón Quirós, Genevieve Buckland, Esther Molina-Montes, María-Dolores Chirlaque, Eva Ardanaz, Pilar Amiano, H Bas Bueno-de-Mesquita, Carla H van Gils, Petra HM Peeters, Nick Wareham, Kay-Tee Khaw, Timothy J Key, Ruth C Travis, Elisabete Weiderpass, Vanessa Dumeaux, Eliv Lund, Malin Sund, Anne Andersson, Isabelle Romieu, Sabina Rinaldi, Paulo Vineis, Melissa A Merritt, Elio Riboli, Rudolf Kaaks

**Affiliations:** 1 Division of Cancer Epidemiology, German Cancer Research Center (DKFZ), Heidelberg, Germany; 2 Institute of Cancer Epidemiology, Danish Cancer Society, 2100 Copenhagen, Denmark; 3 Section of Epidemiology, Department of Public Health, Aarhus University, Aarhus, Denmark; 4 Inserm, Center for Research in Epidemiology and Population Health, U1018, Institut Gustave Roussy, F-94805 Villejuif, France; 5 Paris South University, UMRS 1018, F-94805 Villejuif, France; 6 German Institute of Human Nutrition Potsdam-Rehbruecke Department of Epidemiology Arthur-Scheunert-Allee 114-116, 14558 Nuthetal, Germany; 7 WHO Collaborating Center for Food and Nutrition Policies, Department of Hygiene, Epidemiology and Medical Statistics, University of Athens Medical School, 75 M. Asias Street, Goudi, GR-115 27 Athens, Greece; 8 Hellenic Health Foundation, 10-12 Tetrapoleos Street, GR-115 27 Athens, Greece; 9 Department of Epidemiology, Harvard School of Public Health, 677 Huntington Avenue, Boston, MA 02115 USA; 10 Bureau of Epidemiologic Research, Academy of Athens, 28 Panepistimiou Street, Athens GR-106 79 Greece; 11 Molecular and Nutritional Epidemiology Unit, Cancer Research and Prevention Institute – ISPO, Florence, Italy; 12 Epidemiology and Prevention Unit, Fondazione IRCCS Istituto Nazionale Tumori, Via Venezian, 1, Milan, Italy; 13 Department of Clinical and Experimental Medicine, Federico II University, Naples, Italy; 14 Cancer Registry and Histopathology Unit, “Civile M. P. Arezzo” Hospital, ASP 7 Ragusa, Italy; 15 Center for Cancer Prevention (CPO-Piemonte), Torino, Italy; 16 HuGeF Foundation, Torino, Italy; 17 Public Health and Health Planning Directorate, Asturias, Spain; 18 Unit of Nutrition, Environment and Cancer, Cancer Epidemiology Research Programme, Catalan Institute of Oncology (ICO-IDIBELL), Granada, Spain; 19 Andalusian School of Public Health, Granada, Spain; 20 Consortium for Biomedical Research in Epidemiology and Public Health (CIBER Epidemiología y Salud Pública-CIBERESP), Granada, Spain; 21 Department of Epidemiology, Murcia Regional Health Authority, Murcia, Spain; 22 Navarre Public Health Institute, Pamplona, Spain; 23 Public Health division of Gipuzkoa, Institute BIODonostai, Health Department, Gipuzkoa, Basque Region, France; 24 National Institute for Public Health and the Environment (RIVM), Bilthoven, The Netherlands; 25 Department of Gastroenterology and Hepatology, University Medical Center, Utrecht, The Netherlands; 26 Julius Center for Health Sciences and Primary Care, University Medical Center, 3508, GA, Utrecht, The Netherlands; 27 Department of Epidemiology and Biostatistics, School of Public Health, Faculty of Medicine, Imperial College, London, UK; 28 MRC epidemiology Unit, Cambridge, UK; 29 University of Cambridge, Cambridge, UK; 30 Cancer Epidemiology Unit, University of Oxford, Oxford, UK; 31 Department of Community Medicine, Faculty of Health Sciences, University of Tromsø, Tromsø, Norway; 32 Department of Research, Cancer Registry of Norway, Oslo, Norway; 33 Department of Medical Epidemiology and Biostatistics, Karolinska Institutet, Stockholm, Sweden; 34 Samfundet Folkhälsan, Helsinki, Finland; 35 McGill university, Montreal, Canada; 36 Department of Surgery and Perioperative Sciences, Umea University, Umea, Sweden; 37 Department of Oncology and Radiation Sciences, Oncology, Umeå University Hospital, Umeå, Sweden; 38 Nutritional Epidemiology Group, Section of Nutrition and Metabolism, IARC, Lyon, France; 39 Biomarkers Group, Section of Nutrition and Metabolism, International Agency for Research on Cancer (IARC), Lyon, France; 40 Imperial College School of Public Health, London, UK

**Keywords:** ER-receptor, PR-receptor, Reproductive factors, Risk factors, Menopause, Parity, Oral contraceptive, Breast cancer

## Abstract

**Background:**

The association of reproductive factors with hormone receptor (HR)-negative breast tumors remains uncertain.

**Methods:**

Within the EPIC cohort, Cox proportional hazards models were used to describe the relationships of reproductive factors (menarcheal age, time between menarche and first pregnancy, parity, number of children, age at first and last pregnancies, time since last full-term childbirth, breastfeeding, age at menopause, ever having an abortion and use of oral contraceptives [OC]) with risk of ER-PR- (n = 998) and ER+PR+ (n = 3,567) breast tumors.

**Results:**

A later first full-term childbirth was associated with increased risk of ER+PR+ tumors but not with risk of ER-PR- tumors (≥35 vs. ≤19 years HR: 1.47 [95% CI 1.15-1.88] *p*_trend_ < 0.001 for ER+PR+ tumors; ≥35 vs. ≤19 years HR: 0.93 [95% CI 0.53-1.65] *p*_trend_ = 0.96 for ER-PR- tumors; *P*_*het*_ = 0.03). The risk associations of menarcheal age, and time period between menarche and first full-term childbirth with ER-PR-tumors were in the similar direction with risk of ER+PR+ tumors (p_het_ = 0.50), although weaker in magnitude and statistically only borderline significant. Other parity related factors such as ever a full-term birth, number of births, age- and time since last birth were associated only with ER+PR+ malignancies, however no statistical heterogeneity between breast cancer subtypes was observed. Breastfeeding and OC use were generally not associated with breast cancer subtype risk.

**Conclusion:**

Our study provides possible evidence that age at menarche, and time between menarche and first full-term childbirth may be associated with the etiology of both HR-negative and HR-positive malignancies, although the associations with HR-negative breast cancer were only borderline significant.

## Background

Breast cancer is a complex and heterogeneous disease with a variety of histo-pathological and molecular subtypes with diverse clinical outcomes and relationships with established risk factors [1-3]. The major sub-classification of clinical breast tumors is based on the detection of estrogen (ER) and progesterone (PR) receptors and guides targeted therapies and provides important prognostic information [4]. The presence or absence of hormone receptors, along with human epidermal growth factor-2 (HER2) also broadly correspond to more detailed molecular subclassification of breast tumors, as determined by microarray-based gene expression profiling coupled to hierarchical clustering analyses [5-7]. In addition to the clinical use of ER and PR, epidemiological data indicate that the association of reproductive history with breast cancer differs by the expression of ER and PR receptors [2].

Factors that influence the lifetime cumulative exposure to hormones during reproductive life, such as the age at menarche, age at first child-birth, time between age at menarche and first child birth, number of children, use of oral contraceptives (OC) and breastfeeding, have been suggested to be associated with risk of hormone receptor (HR)-positive malignancies (ER-positive or joint ER+PR+)[2,8-11]. However, distinct risk factors for HR-negative (ER-negative, or joint ER-PR) cancer are debated [2,3,8,12] and the etiologies of ER+PR+ and ER-PR- tumors remain unclear.

The incidence of HR-negative disease drops remarkably after menopause [13] suggesting that ovarian derived sex steroid hormones do have an impact on HR-negative tumors and recent studies are starting to show risk associations of reproductive factors with HR-negative malignancies [2,3]. In fact, opposite risk associations between ER-PR- and ER+PR+ tumors have been observed with parity [11,14], age at first pregnancy [9] and breastfeeding[11]. Nonetheless, due to the rarity and heterogeneous nature of HR-negative breast tumors, epidemiological studies have been hindered by small sample sizes resulting in inconsistent risk associations between reproductive factors and HR-negative disease [8,11,15,16].

The incidence of HR-negative disease drops remarkably after menopause [13] suggesting that ovarian derived sex steroid hormones do have an impact on HR-negative tumors and recent studies are starting to show risk associations of reproductive factors with HR-negative malignancies [2,3]. In fact, opposite risk associations between ER-PR- and ER+PR+ tumors have been observed with parity [11,14], age at first pregnancy [9] and breastfeeding [11]. Nonetheless, due to the rarity and heterogeneous nature of HR-negative breast tumors, epidemiological studies have been hindered by small sample sizes resulting in inconsistent risk associations between reproductive factors and HR-negative disease [3,8,14,15].

## Methods

The European Prospective Investigation into Cancer and Nutrition (EPIC) is a multi-center prospective cohort study designed to investigate the relationships between diet, nutrition and metabolic factors and cancer, consisting of approximately 360,000 women and 150,000 men aged mostly between 25–70 years [16,17]. All participants were enrolled between 1992 and 2000 and came from 23 regional and national research centers located in 10 western European countries: Denmark, France, Italy, Germany, Greece Norway, Spain, Sweden, The Netherlands and the United Kingdom. Extensive details about the standardized procedures for recruitment, measuring baseline anthropometry, questionnaires on current habitual diet, reproductive and menstrual history, exogenous hormone use [OC and hormone replacement therapy (HRT) use], medical history, lifetime smoking and alcohol consumption history, occupation, level of education and physical activity and biological sample collection at study centers are given elsewhere [16,17]. All subjects gave written informed consent. The Internal Review Boards of the International Agency for Research on Cancer and the local ethics committees in participating countries approved the analyses based on EPIC participants.

### Study participants

Of the approximately 360,000 female participants in EPIC, women were excluded *a priori* if they had a history of cancer prior to recruitment or were missing a diagnosis or censoring date, thus leaving 345,153 participants. At the time of this analysis, three EPIC study centers, (Granada, Murcia and Malmo), did not provide any information on breast tumor hormone receptor status and therefore were excluded from this analysis (n = 26,091). Women were further excluded if they were missing questionnaire data (n = 526) or were missing data on age at menarche, age at menopause, age at first full-term birth, ever use of OCs, number of full-term births, age at last full-term childbirth and duration of breastfeeding (n = 7,439). This left a total of 311,097 women with 9,456 first primary invasive breast cancer cases from 10 countries for the present analysis.

### Questionnaire data and classification of reproductive variables

The details of standardized procedures for collecting baseline information on the age at first and last menstruation, parity, breastfeeding, exogenous hormone use, and hysterectomy from the general lifestyle questionnaire has been previously reported [17,18]. Briefly, in Greece, Italy, the Netherlands, Sweden and the United Kingdom, age at menarche was asked in years. In the other countries, age at menarche was asked in defined categories (≤8, 9, 10,…, 18, 19 or >19 years). The number of full-term pregnancies (defined as the sum of all live and stillborn children born) and spontaneous or induced abortions were also collected at baseline, together with the ages of the first three and last deliveries and the ages at first and last induced or spontaneous abortions and stillbirths. Except for Norway and the Swedish center Umeå, where information about multiple pregnancies was available, the number of pregnancies is overestimated as multiple pregnancies were counted as different pregnancies. The length of time between menarche and age at first pregnancy was estimated among women who had menarche between the ages of 8 and 20 years (time between menarche and first full-term birth = age at first full-term birth – age at menarche).

Women were considered postmenopausal at recruitment if they had had no menstrual cycles in the last 12 months, were older than 55 years (if the menstrual cycle history was missing), or had a bilateral oophorectomy. Women who were aged 46–55 years and had incomplete or were missing questionnaire data on menstrual history were classified with a peri/-or of unknown menopausal status. Women were deemed premenopausal if they reported regular menstrual cycles in the last 12 months or if they were younger than 46 years of age (if the menstrual cycle history was missing).

The details of standardized procedures for measuring height and weight at EPIC study centers has also been previously reported [19]. In most countries, height, weight and waist and hip circumferences were measured to the nearest centimeter and kilogram, in light clothing, according to standardized protocols. In Norway, Umeå and a large proportion from France, subjects’ height and weight were measured and self-reported by the cohort participants themselves, following detailed instructions [17,19]. For subjects that had neither self-reported nor measured weight or height data, the center-, age- and gender-specific average weight and height values were imputed for anthropometry variables used for adjustment purposes only. A sensitivity analysis that restricted the adjusted variables to those without imputation showed similar results to those presented (data not shown).

### Prospective ascertainment of breast cancer cases and the coding of receptor status

In all countries (except for France, Germany and Greece) incident breast cancer cases were identified using record linkage with cancer and pathology registries. In France, Germany and Greece, cancer occurrence was prospectively ascertained through linkage with health insurance records and regular direct contact with participants and their next of kin, and all reported breast cancer cases were then systematically verified against clinical and pathological records. Mortality data were coded according to the 10th Revision of the International Statistical Classification of Diseases, Injuries, and Causes of Death (ICD-10), and cancer incidence data were coded according to the International Classification of Diseases for Oncology (ICD-O-2). Invasive (primary, malignant) breast cancer cases were classified as per the International Classification of Diseases for Oncology (Topography C50), second revision (ICD-O-2). Breast tumor receptor status was standardized across EPIC centers using the following criteria for a positive expression: ≥10% cells stained, any ‘plus-system’ description, ≥20 fmol/mg, an Allred score of ≥3, an IRS ≥2, or an H-score ≥10 [20].

Vital status was collected from regional or national mortality registries. The last updates of endpoint data for cancer incidence and vital status were between 2005 and 2010, depending on the center. Women were considered at risk from the time of recruitment until breast cancer diagnosis or censoring (age at death, loss to follow up, end of follow up, or diagnosis of other cancer) respectively. A total of 7,095 breast cancer cases had information on ER status (5,723 ER-positive, 1,372 ER-negative); of which, 5,843 had further information on PR status (3,567 ER+PR+, 1,078 ER+PR-, 200 ER-PR+, 998 ER-PR-).

### Statistical analysis

Associations between reproductive factors and the risk of breast cancer subtype were evaluated using Cox proportional hazards models to estimate hazard ratios (HR) and 95% confidence intervals (CIs). Breast cancer subtypes were defined as jointly classified ER+PR+ or ER-PR- breast tumors. Results for ER-positive versus ER-negative (ignoring PR status); and PR-positive versus PR-negative (ignoring ER status) were generally similar to the jointly defined ERPR breast cancer subtypes and have been included in Additional file 1: Table S1. Results for breast tumors with discordant ER and PR status and unknown ER and/or PR status have been reported in Additional file 2: Table S2. All analyses were stratified by age at recruitment in one-year categories and by study center, to prevent violations of the proportional-hazard assumption. Trend tests across levels of exposure categories were performed on the continuous categorical variables entered as ordered, quantitative variables into the models.

Age at menarche was categorized as ≤12, 13–14 and ≥15 years and time between menarche and first full-term childbirth as <10 and ≥10 years. Parity related variables were divided into the following categories ever vs. never, number of full-term pregnancies (1, 2, ≥3), age at first full-term childbirth as ≤19, 20–24, 25-29,30-34, ≥35 years, age at last full-term childbirth since recruitment as ≤24, 25–29, 30-34, ≥35 years and time since last child birth as ≤20 and >20 years. Breastfeeding was categorized as ever versus never, and ≤1 month, 2-3, 4–6, 7–12, 13–17 and ≥18 months for total cumulative duration of breastfeeding. Dichotomized categories of ever vs. never having had a spontaneous or induced abortion, ever vs. never OC use, and current versus not currently using OCs (at baseline) also were analyzed. The duration of OC use was categorized into ≤1, 2–4, 5–9, and ≥10 years. Age at menopause was divided into the categories ≤48, 49–50, 51–54 and ≥55 years.

A basic model stratified by age and center and a multivariable model further adjusted for body mass index (BMI kg/m^2^, as a continuous variable), height (as a continuous variable), menopausal status at enrolment (premenopausal, peri-/unknown menopausal, postmenopausal [natural and surgical menopause], HRT use (premenopausal, ever use, never use and missing in postmenopausal women only), smoking status (current, former, never, missing), baseline alcohol consumption (non-consumers, 0.1–6 g/day, 6-12 g/day, 12-24 g/day, 24-60 g/day and greater than 60 g/day, missing), physical activity (Cambridge Index: active, moderately active, moderately inactive and inactive, missing [21]), education level (none, primary school, technical/professional school, secondary school, longer education including university degree, missing) were assessed. Missing values (generally <2%) were accounted for by creating an extra category in each covariate.

To avoid collinearity when studying the joint effect of the number of full-term pregnancies, age at first and last full-term childbirth and time since last childbirth, we used the approach described by *Heuch et al.*[22]. In analyses including age at last full-term childbirth and time since last childbirth in an age adjusted model, the general age effect was represented by the age effect among nulliparous women. We assigned constant values for age at full-term childbirth and time since last full-term childbirth (corresponding to the reference categories) to nulliparous women, and indicator variables were introduced in the model to ensure that the risk estimates reflected effects in parous women only.

Differences in risk estimates of a given factor and across breast cancer subtypes were analyzed using the data augmentation method as described by Lunn and McNeil, using a likelihood ratio test to compare the model with and without interaction terms between the exposure of interest and breast cancer subtype [23]. Women were considered at risk of a given breast cancer subtype until they were diagnosed with a different competing breast cancer subtype or were diagnosed with breast cancer and the receptor status information was missing. These women were censored at the time of occurrence of the competing breast cancer subtype [23]. To assess whether breast cancer subtype reproductive risk factors changed across women after menopause, left and/or right side censoring was used to count person years within defined age periods <50 years, and ≥50 years. As no differences were observed between risk estimates of reproductive risk factors and breast cancer subtype risk across the age-bands we report results for all women combined. A sensitivity analysis restricting to cases with any indication of an ER and PR expression versus a complete absence of ER and PR expression (0% cells stained, a “-“ description (i.e. a negative/minus symbol description), 0 fmol/mg, an Allred score of 0, an IRS = 0, or an H-score = 0) was also performed. Heterogeneity in the risk associations between subgroups by age at diagnosis (<50 vs. ≥50 years), OC use, center, median BMI, age at first pregnancy and ever having breastfed were also examined using the Cochran’s Q statistic. A previous analysis on postmenopausal HRT use has been reported in the EPIC cohort, therefore HRT use as a predictor of breast cancer risk by HR status was not included in this analysis [24]. All statistical analyses were performed using the SAS software package, version 9.2 (SAS Institute, Cary, NC).

## Results

A total cohort of 311,097 women was followed for a sum of 3,346,356 person years. Women were recruited into the EPIC study at the median age of 51.1 years (Table 1). At the time of recruitment, 46.5% of the women were postmenopausal and the median age of menopause was 50.0 years. For parous women, the median age at first full-term birth across the EPIC centers was 24.0 and the median time between menarche and first child birth was 11.0 years. The median age at last full-term pregnancy was 29.0 years, and a median time of 22.9 years had passed since the last childbirth. Of the women who had breastfed (83.8%), the median time of breastfeeding was 6.0 months for all pregnancies.

**Table 1:** Baseline characteristics of 311,097 EPIC cohort participants

**Study variables**	**All women**
	**(n = 311,097)**
Age at recruitment (median, range)	51.1 (19.9-98.5)
Age end of follow-up (1st tumor) (median, range)	62.0 (21.2-102.4)
Years of follow up (median, range)	11.3 (0.0-16.7)
**Menopausal status**	
Premenopausal	108,084 (34.7)
Peri/Unknown	58,345 (18.8)
Postmenopausal	144,668 (46.5)
Age at menarche (median, range)	13.0 (8.0-20.0)
Age at menopause (median, range)^1^	50.0 (16.0-67.0)
Ever full-term pregnancy (yes, %)	255,877 (84.5)
Age at first full-term pregnancy (median, range)^2^	24.0 (12.0-56.0)
Time between menarche and first pregnancy (years) (median, range)^2^	11.0 (0.0-41.0)
**Number of full-term pregnancies**^2^	
1 child	46,572 (18.8)
2 children	122,590 (49.4)
≥3 children	78,983 (31.8)
Breastfeeding (yes, %)^2^	200,885 (83.8)
Duration of breastfeeding, all pregnancies (months) (median, range)^2,3^	6.0 (0.2-286.4)
Age at last full-term pregnancy (median, range)^2^	29.0 (13.0-56.0)
Time since last full-term pregnancy (years) (median, range)^2^	22.9 (0.0-69.1)
Ever had an abortion (yes, %)^4^	87,130 (40.1)
**Oral contraceptive (OC) use**	
Never OC user	125,229 (41.4)
Past OC user	160,926 (53.2)
Current OC user	16,560 (5.5)
Age when you started using the pill (median, range)^5^	24.0 (8.0-50.0)
Duration of using the pill (years) (median, range)^5^	5.0 (1.0-15.0)

^1^In natural and surgically menopausal women only.

^2^In parous women only.

^3^In women who breast-fed only.

^4^Both spontaneous and induced abortions.

^5^In women who ever used OC.

A statistically significant heterogeneity between the risk of ER-PR- (n = 998) and ER+PR+ (n = 3,567) tumors was observed for age at first full-term childbirth (Table 2). While Cox regression models showed no association with risk of ER-PR-malignancies, a first full-term childbirth after the age of 35 was associated with an increased risk of ER+PR+ tumors (≥35 vs. ≤19 years HR: 1.47 [95% CI 1.15-1.88] *p*_trend_ < 0.001, *P*_*het*_ = 0.03).

**Table 2:** Reproductive factors and risk of ER+PR+ and ER-PR- breast cancer in all women

	**Age and center stratified**						**Multivariable adjusted**^**1**^					
	**ER+PR+**			**ER-PR-**			**ER+PR+**			**ER-PR-**		
	**(n = 3567)**			**(n = 998)**			**(n = 3567)**			**(n = 998)**		
**Reproductive factor**	**Cases**	**HR**	**95% CI**	**Cases**	**HR**	**95% CI**	**Cases**	**HR**	**95% CI**	**Cases**	**HR**	**95% CI**
**Age at menarche**												
<13 years	1373	1.00	Reference	376	1.00	Reference	1373	1.00	Reference	376	1.00	Reference
14 years	1717	0.96	(0.90-1.04)	481	0.98	(0.86-1.13)	1717	0.96	(0.89-1.03)	481	0.97	(0.85-1.12)
≥15 years	439	0.76	(0.68-0.85)	135	0.85	(0.69-1.04)	439	0.76	(0.68-0.85)	135	0.84	(0.69-1.03)
P for trend			<0.001			0.17			<0.001			0.17
Subtype heterogeneity^*2*^						0.44						0.48
**Age at menopause**^**3**^												
≤48 years	464	1.00	Reference	135	1.00	Reference	464	1.00	Reference	135	1.00	Reference
49–50 years	397	1.10	(0.95-1.26)	116	1.09	(0.84-1.41)	397	1.12	(0.98-1.29)	116	1.09	(0.84-1.42)
51–54 years	280	1.04	(0.89-1.21)	63	0.88	(0.64-1.20)	280	1.06	(0.91-1.24)	63	0.87	(0.64-1.20)
≥55 years	121	1.19	(0.97-1.47)	32	1.06	(0.71-1.58)	121	1.17	(0.95-1.44)	32	1.03	(0.69-1.54)
P for trend			0.18			0.79			0.18			0.79
Subtype heterogeneity^*2*^						0.40						0.34
**Ever a full-term birth**												
No	446	1.00	Reference	115	1.00	Reference	446	1.00	Reference	115	1.00	Reference
Yes	2994	0.86	(0.77-0.95)	843	0.97	(0.80-1.19)	2994	0.87	(0.78-0.96)	843	0.98	(0.80-1.20)
P for significance			0.003			0.78			0.01			0.78
Subtype heterogeneity^*2*^						0.92						0.88
**Number of full-term childbirths**^**4**^												
1 child	612	1.00	Reference	160	1.00	Reference	612	1.00	Reference	160	1.00	Reference
2 children	1497	0.92	(0.84-1.01)	432	1.02	(0.85-1.22)	1497	0.92	(0.84-1.01)	432	1.01	(0.84-1.22)
>3 children	840	0.77	(0.69-0.85)	244	0.89	(0.72-1.09)	840	0.76	(0.68-0.85)	244	0.89	(0.73-1.10)
P for trend			<0.001			0.19			<0.001			0.19
Subtype heterogeneity^*2*^						0.22						0.20
**Age at first full-term childbirth**^**4**^												
≤19 years	357	1.00	Reference	103	1.00	Reference	357	1.00	Reference	103	1.00	Reference
20–24 years	1369	1.06	(0.94-1.20)	431	1.20	(0.97-1.50)	1369	1.07	(0.95-1.21)	431	1.20	(0.96-1.50)
25–29 years	912	1.20	(1.06-1.36)	242	1.16	(0.92-1.47)	912	1.22	(1.07-1.39)	242	1.17	(0.92-1.50)
30–34 years	275	1.35	(1.15-1.59)	61	1.10	(0.80-1.52)	275	1.39	(1.18-1.64)	61	1.12	(0.81-1.56)
≤35 years	82	1.44	(1.13-1.83)	14	0.91	(0.52-1.60)	82	1.47	(1.15-1.88)	14	0.93	(0.53-1.65)
P for trend			<0.001			0.96			<0.001			0.96
Subtype heterogeneity^*2*^						0.02						0.03
**Time between menarche and first full-term childbirth**^**4**^												
<10 years	837	1.00	Reference	257	1.00	Reference	837	1.00	Reference	257	1.00	Reference
≥10 years	2133	1.21	(1.12-1.31)	592	1.14	(0.98-1.32)	2133	1.22	(1.12-1.33)	592	1.15	(0.99-1.34)
P for significance			<0.001			0.09			<0.001			0.09
Subtype heterogeneity^*2*^						0.49						0.52
**Age at last full-term childbirth**^**4**^												
≤24 years	463	1.00	Reference	138	1.00	Reference	463	1.00	Reference	138	1.00	Reference
25–29 years	1058	0.98	(0.88-1.10)	321	1.01	(0.82-1.23)	1058	0.98	(0.88-1.10)	321	1.01	(0.82-1.23)
30–34 years	984	1.07	(0.95-1.20)	263	0.97	(0.78-1.19)	984	1.07	(0.96-1.20)	263	0.98	(0.79-1.21)
≥35 years	490	1.11	(0.97-1.26)	130	1.01	(0.79-1.29)	490	1.12	(0.98-1.28)	130	1.03	(0.80-1.33)
P for trend			0.03			0.90			0.02			0.90
Subtype heterogeneity^*2*^						0.24						0.29
**Time since last full-term childbirth**^**4**^												
≤20 years	1043	1.00	Reference	300	1.00	Reference	1043	1.00	Reference	300	1.00	Reference
>20 years	1952	0.87	(0.78-0.96)	552	1.00	(0.82-1.22)	1952	0.86	(0.77-0.95)	552	0.98	(0.81-1.20)
P for significance			0.01			0.98			0.01			0.98
Subtype heterogeneity^*2*^						0.20						0.24
**Ever breast-fed**^**4**^												
No	538	1.00	Reference	152	1.00	Reference	538	1.00	Reference	152	1.00	Reference
Yes	2317	0.99	(0.90-1.09)	642	0.97	(0.81-1.16)	2317	0.99	(0.89-1.09)	642	0.98	(0.81-1.17)
P for significance			0.82			0.74			0.76			0.74
Subtype heterogeneity^*2*^						0.85						0.93
**Total cumulative breastfeeding duration**^**4,5**^												
<1 month	249	1.00	Reference	72	1.00	Reference	249	1.00	Reference	72	1.00	Reference
1–3 months	602	1.04	(0.89-1.20)	155	0.91	(0.69-1.21)	602	1.04	(0.89-1.20)	155	0.91	(0.69-1.21)
4–6 months	460	0.97	(0.83-1.14)	137	0.98	(0.74-1.32)	460	0.97	(0.83-1.14)	137	0.99	(0.74-1.32)
7–12 months	487	0.97	(0.83-1.13)	132	0.91	(0.68-1.22)	487	0.97	(0.83-1.13)	132	0.91	(0.68-1.23)
13–17 months	182	0.91	(0.75-1.11)	62	1.10	(0.78-1.57)	182	0.92	(0.75-1.12)	62	1.12	(0.79-1.60)
≥18 months	293	1.09	(0.91-1.31)	77	1.04	(0.73-1.46)	293	1.11	(0.92-1.33)	77	1.07	(0.75-1.51)
P for trend			0.99			0.54			0.90			0.54
Subtype heterogeneity^*2*^						0.58						0.53
**Ever an abortion**^**6**^												
No	1552	1.00	Reference	435	1.00	Reference	1552	1.00	Reference	435	1.00	Reference
Yes	1016	1.00	(0.92-1.08)	293	1.00	(0.86-1.16)	1016	0.99	(0.91-1.07)	293	1.00	(0.86-1.16)
P for significance			0.97			0.98			0.72			0.98
Subtype heterogeneity^*2*^						0.97						0.86
**OC use at recruitment**												
Never OC user	1477	1.00	Reference	379	1.00	Reference	1477	1.00	Reference	379	1.00	Reference
Past OC user	1839	1.00	(0.93-1.08)	548	1.11	(0.96-1.28)	1839	0.97	(0.90-1.05)	548	1.09	(0.94-1.26)
Current OC user	108	1.20	(0.97-1.47)	33	1.08	(0.73-1.59)	108	1.19	(0.96-1.47)	33	1.09	(0.74-1.63)
P for trend			0.42			0.20			0.95			0.20
Subtype heterogeneity^*7*^						0.34						0.30
**Age started OC**^**8**^												
≤24 years	899	1.00	Reference	292	1.00	Reference	899	1.00	Reference	292	1.00	Reference
25–29 years	335	0.86	(0.75-0.99)	94	0.86	(0.66-1.11)	335	0.87	(0.76-1.00)	94	0.86	(0.66-1.11)
30–34 years	303	0.91	(0.78-1.07)	90	0.92	(0.69-1.23)	303	0.93	(0.79-1.08)	90	0.92	(0.69-1.22)
≥35 years	265	1.07	(0.90-1.28)	73	0.97	(0.70-1.36)	265	1.09	(0.91-1.30)	73	0.97	(0.70-1.36)
P for trend			0.83			0.75			0.67			0.75
Subtype heterogeneity^*2*^						0.70						0.61
**Duration of OC use**^**8**^												
1 year or less	396	1.00	Reference	114	1.00	Reference	396	1.00	Reference	114	1.00	Reference
2–4 years	450	1.02	(0.89-1.17)	116	0.86	(0.66-1.12)	450	1.03	(0.89-1.18)	116	0.86	(0.67-1.12)
5–9 years	426	1.06	(0.92-1.22)	122	0.97	(0.75-1.26)	426	1.06	(0.92-1.22)	122	0.97	(0.75-1.27)
≥10 years	521	1.02	(0.89-1.17)	187	1.11	(0.87-1.41)	521	1.02	(0.89-1.18)	187	1.11	(0.87-1.42)
P for trend			0.70			0.21			0.68			0.21
Subtype heterogeneity^*2*^						0.36						0.37

^1^Stratified by age at recruitment and center and further adjusted for BMI, height, menopausal status at enrolment, HRT use, physical activity, smoking status, alcohol consumption and attained level of education; ^2^heterogeneity between ER+PR+ and ER-PR- tumors was assessed on the trend score using the data augmentation method as described by Lunn and McNeil; ^3^in postmenopausal women only; ^4^in parous women only; ^5^in women who breast-fed only; ^6^in both spontaneous and induced abortions; ^7^heterogeneity between ER+PR+ and ER-PR- tumors was assessed on the unordered categorical variable of never, past and current OC use using the data augmentation method as described by Lunn and McNeil; ^8^in women who ever used OC.

For ER-PR- breast tumors, similar risk associations to ER+PR+ breast tumors were observed with increasing menarcheal age *(≥15 vs. ≤ 13 years* ER-PR- HR: 0.84 [95% CI 0.69-1.03], *P*_*trend*_ =0.17; ER+PR+ HR: 0.76 [95% CI 0.68-0.85], *P*_*trend*_ <0.001; *P*_*het*_ = 0.48), and among parous women, with longer time between menarche and first full-term childbirth *(≥10 vs. < 10 years* ER-PR- HR: 1.15 [95% CI 0.99-1.34], *P*_*trend*_ =0.09; ER+PR+ HR: 1.22 [95% CI 1.12-1.33], *P*_*trend*_ <0.001; *P*_*het*_ = 0.52). Although the relative risk estimates for ER-PR- breast malignancies were in a similar direction to the ER+PR+ tumors and no statistical heterogeneity between the breast cancer subtypes was observed, it should be noted that risk associations for ER-PR- malignancies were weaker in magnitude and failed to reach statistical significance.

Ever a full-term childbirth, number of childbirths, age- and time since last full-term childbirth were generally not associated with the risk of ER-PR- malignancies but were associated with ER+PR+ tumors, however no statistical heterogeneity between the breast cancer subtypes was observed. Moreover, OC use, duration of OC use, breast feeding, ever having an abortion (spontaneous or induced) and age at menopause showed no significant associations with either ER-PR- or ER+PR+ breast cancer.

When all of the pregnancy related variables were examined together in the same model, risk associations for an increased number of full-term births, and later first and last full-term childbirth with ER-PR- tumors appeared to slightly strengthen; however they remained statistically not significant and had wider confidence intervals (Table 3). When analyses were restricted to postmenopausal women, the risk estimates for a later age at first full-term childbirth with ER+PR+ tumors were stronger (≥35 vs. ≤19 years HR: 1.64 [95% CI 1.05-2.56] *p*_trend_ = 0.002) compared to the estimates for all the women combined (≥35 vs. ≤19 years HR: 1.30 [95% CI 0.95-1.79] *p*_trend_ = 0.02).

**Table 3:** A mutually adjusted model of pregnancy related variables and risk of ER+PR+ vs. ER-PR- breast cancer

	**Age and center stratified**				**Multivariable adjusted**^**1**^				**Postmenopausal women**^**2**^			
	**ER+PR+**		**ER-PR-**		**ER+PR+**		**ER-PR-**		**ER+PR+**		**ER-PR-**	
	**(n = 3387)**		**(n = 944)**		**(n = 3387)**		**(n = 944 )**		**(n = 1755)**		**(n = 477)**	
**Reproductive factor**	**HR**	**95% CI**	**HR**	**95% CI**	**HR**	**95% CI**	**HR**	**95% CI**	**HR**	**95% CI**	**HR**	**95% CI**
**Number of full-term childbirths**^**3**^												
1 child	1.00	Reference	1.00	Reference	1.00	Reference	1.00	Reference	1.00	Reference	1.00	Reference
2 children	0.93	(0.84-1.03)	0.97	(0.80-1.19)	0.93	(0.84-1.03)	0.98	(0.80-1.19)	0.96	(0.83-1.10)	0.90	(0.69-1.19)
≥3 children	0.77	(0.68-0.87)	0.83	(0.66-1.05)	0.77	(0.68-0.87)	0.84	(0.66-1.06)	0.79	(0.67-0.93)	0.90	(0.65-1.22)
P for trend		<0.001		0.09		<0.001		0.10		0.002		0.54
Subtype heterogeneity^*4*^				0.53				0.47				0.22
**Age at first full-term childbirth**^**3**^												
≤19 years	1.00	Reference	1.00	Reference	1.00	Reference	1.00	Reference	1.00	Reference	1.00	Reference
20–24 years	1.05	(0.92-1.19)	1.11	(0.88-1.40)	1.05	(0.92-1.19)	1.10	(0.87-1.39)	1.17	(0.97-1.40)	1.14	(0.82-1.59)
25–29 years	1.16	(0.99-1.35)	0.96	(0.72-1.28)	1.16	(0.99-1.36)	0.96	(0.72-1.28)	1.32	(1.05-1.64)	1.02	(0.68-1.54)
30–34 years	1.24	(1.00-1.53)	0.83	(0.55-1.25)	1.24	(1.00-1.53)	0.83	(0.55-1.25)	1.57	(1.16-2.11)	0.88	(0.49-1.57)
≥35 years	1.32	(0.96-1.82)	0.69	(0.35-1.36)	1.30	(0.95-1.79)	0.69	(0.35-1.36)	1.64	(1.05-2.56)	0.81	(0.32-2.03)
P for trend		0.02		0.19		0.02		0.19		0.002		0.51
Subtype heterogeneity^*4*^				0.03				0.03				0.05
**Age at last full-term childbirth**^**3**^												
Less than 25	1.00	Reference	1.00	Reference	1.00	Reference	1.00	Reference	1.00	Reference	1.00	Reference
Between 25 and 30	0.90	(0.80-1.02)	1.15	(0.93-1.44)	0.90	(0.80-1.02)	1.16	(0.93-1.44)	0.87	(0.74-1.02)	1.13	(0.83-1.53)
Between 30 and 35	0.97	(0.83-1.13)	1.33	(1.00-1.77)	0.97	(0.84-1.13)	1.33	(1.00-1.77)	0.85	(0.69-1.04)	1.27	(0.86-1.86)
Greater than 35	0.89	(0.72-1.09)	1.29	(0.88-1.90)	0.89	(0.73-1.10)	1.30	(0.88-1.91)	0.83	(0.63-1.10)	1.39	(0.83-2.34)
P for trend		0.53		0.10		0.58		0.09		0.18		0.18
Subtype heterogeneity^*4*^				0.17				0.17				0.09
**Time since last full-term childbirth**^**3**^												
≤20 years	1.00	Reference	1.00	Reference	1.00	Reference	1.00	Reference	1.00	Reference	1.00	Reference
>20 years	0.87	(0.77-0.98)	1.01	(0.81-1.26)	0.86	(0.77-0.97)	1.00	(0.80-1.25)	0.90	(0.73-1.12)	1.10	(0.74-1.65)
P for significance		0.02		0.94		0.02		0.99		0.35		0.63
Subtype heterogeneity^*4*^				0.24				0.24				0.29

^1^Stratified by age at recruitment and center and further adjusted for BMI, height, menopausal status at enrolment, HRT use, physical activity, smoking status, alcohol consumption and attained level of education; ^2^stratified by age at recruitment and center and further adjusted for BMI, height, HRT use, physical activity, smoking status, alcohol consumption and attained level of education; ^3^mutually adjusted for the pregnancy related variables in this table; ^4^heterogeneity between ER+PR+ and ER-PR- tumors was assessed on the trend score using the data augmentation method as described by Lunn and McNeil.

Subgroup analyses showed no heterogeneity in most of the risk estimates of the reproductive variables for ER-PR- and ER+PR+ tumors (data not shown), with the exception of the duration of breastfeeding by age at first birth among parous women (P_heterogeneity_ = 0.002). Among women who had their first full-term childbirth before the age of 25, a longer duration of total breastfeeding showed an indication of a decreased risk association with ER+PR+ tumors (≥18vs. ≤ 1 month HR: 0.86 [95% CI 0.67-1.10], P_trend_ = 0.01). In contrast, among women who had their first full-term childbirth after the age of 25, a longer cumulative duration of breastfeeding was associated with a particular increased risk of ER+PR+ tumors (≥18vs. ≤ 1 month HR: 1.50 [95% CI 1. 13–1.99], P_trend_ = 0.005). For ER-PR- tumors, risk estimates in the similar direction to the ER+PR+ tumors were observed, however statistically not significant (first childbirth before the age of 25, ≥18vs. ≤ 1 month of breast feeding HR: 0.90 [95% CI 0.58-1.39], P_trend_ = 0.89; first childbirth after the age of 25, ≥18vs. ≤ 1 month of breast feeding HR: 1.31 [95% CI 0.72-2.39], P_trend_ = 0.41).

In the sensitivity analysis restricted to cases with any indication of a positive ER and PR expression versus a complete absence of ER and PR expression with reproductive factors showed similar patterns with risk of joint ER+PR+ and ER-PR- breast cancer subtypes (data not shown).

## Discussion

Our results showed a significantly heterogeneous risk association for age at first full-term pregnancy by receptor status of the tumor, where later first full-term childbirth was associated with increased risk of ER+PR+ tumors but not with risk of ER-PR- tumors. Menarcheal age and time period between menarche and first full-term childbirth showed suggestively similar risk associations with both ER-PR- and ER+PR+ tumors, however the risk estimates for ER-PR- tumors were generally weaker than for their ER+PR+ counterparts and only borderline significant. Although the heterogeneity between breast cancer subtypes was not statistically significant, other parity related factors (such as ever having a full-term childbirth, number of full-term childbirths, age- and time since last full-term childbirth) were associated only with ER+PR+ malignancies. Finally, the factors related to breastfeeding and OC use were generally not associated with HR-positive or HR-negative breast cancer risk.

Previous prospective studies investigating the association of reproductive factors with HR-positive breast cancer have shown relatively consistent inverse risk associations with increasing menarcheal age, ever having a full-term childbirth and particularly a full-term childbirth at an early age [3,9,14,25]. However, consensus on the associations with HR-negative tumors has not been reached because previous prospective studies have lacked sufficient sample sizes [3,9,26] and because of the heterogeneous nature of HR-negative subtypes [1]. A more recent study within the Women’s Health Initiative [14] showed that ever having a full-term childbirth was associated with an increased risk of triple-negative breast cancer (ER-, PR- and HER2-) (n = 307) and the positive association was strengthened with an increasing number of full-term births. We were unable to confirm the positive risk association with ever having a full-term childbirth and increasing number of full-term pregnancies with HR-negative tumors, however we did not have information on triple negative tumors.

We observed for both ER-PR- and ER+PR+ tumors, similar risk associations with increasing menarcheal age and a longer time between menarche and first full-term childbirth. A recent study within the EPIC cohort previously reported on the association of menarche with both ER-PR- and ER+PR+ tumors within the context of childhood growth and earlier sexual maturity [27]. This study extends onto this study of menarche and growth and focuses on the time period between sexual maturity and first full-term pregnancy, thus illustrating the complex and entwined nature of endocrine-sensitive tumors with hormones during different life phases of growth, sexual maturity and reproduction. The inverse association of menarcheal age with increased breast cancer risk is thought to be resultant of a longer exposure to estrogens during a women’s reproductive life [2] but may also reflect early pubertal years characterized by more intensive and increased exposure to estrogen [27,28]. Estrogens have been long established to have a late-stage growth promoting effect on estrogen sensitive tumors [29], however, evidence suggests that estrogens may also play an important role in earlier developmental stages of both HR-positive and -negative tumor types [30]. Mammary stem cells have been shown to respond to sex steroid hormones without having a clear expression of an ER or PR [31]. Further, the EPIC cohort, showed that pre-diagnostic levels of estrogens were associated with both HR-negative and HR-positive postmenopausal breast cancer [20]. The longer time between menarche and a women’s first full-term childbirth would equate to a longer period of time with undifferentiated breast epithelial tissue and a shorter period of time that the breast is resistant to malignant transformation [26] and thus may have etiological importance in the formation of ER-PR- tumors as well.

We observed a significantly different risk association for a later age at first full-term childbirth with risk of HR-negative and HR-positive tumors, whereas the associations for parity related factors (such as ever having a full-term childbirth, age a last full-term childbirth and time since last full-term childbirth) appeared to aggregate around HR-positive tumors. The role of a pregnancy with the risk of breast cancer is thought to stem from two major avenues, firstly, hormonal changes before and after pregnancy and secondly [26], dramatic structural changes in the ductal system of the breast after pregnancy [26,32]. A full-term childbirth is associated with a long term post-pregnancy reduction in levels of circulating hormones [26]. Before a women’s first pregnancy the breast contains a high proportion of undifferentiated ducts and associated alveolar buds. Complete differentiation only occurs during pregnancy and lactation via complex morphological, physiological, and molecular changes [32]. Terminally differentiated epithelial cells are more resistant to carcinogenic influences because of lower proliferation rates and longer DNA repair phases [26]. The distinct inverse risk association for an earlier age at first full-term childbirth with ER+PR+ disease could be due to a shorter exposure to higher levels of ovarian estrogens and a shorter period of time of undifferentiated breast epithelial cells.

Recent prospective studies have reported reduced risk associations with breastfeeding with both ER-PR- and ER+PR+ breast cancer [3,14]. In the current analysis, we also observed an inverse risk association for both ER-PR- and ER+PR+ malignancies with a longer cumulative duration of breastfeeding however, this was restricted to women who had an early full-term childbirth. In contrast to the recent studies, among women who had a later first full-term childbirth, an increased risk with ER-PR- and ER+PR+ breast tumors with a longer total duration of breastfeeding was observed. Breastfeeding is thought to protect a woman from developing breast cancer by increasing breast differentiation, postponing the return of the ovulatory menstrual cycle post-pregnancy, and/or changing the hormonal environment of the breast [9,26,32]. The inverse risk association of HR-negative and HR-positive tumors with breastfeeding coupled with a longer duration among young first time mothers could convey a similar protection. In addition, lactation at a younger age would also mean a shorter period of undifferentiated breast epithelial tissue [26].

OC use has been extensively studied by many epidemiological studies and most studies have found either no association or a moderate increased risk of overall breast cancer, particularly among very young women and recent OC users [26]. More recent case–control studies investigating the risk of HR-defined breast cancer have started showing relationships of OC use with HR-negative breast cancer [33-37]. In the current study, we were unable to confirm significant risk associations of ever OC use and a longer duration of OC use with HR-negative tumors. There were only a small number of baseline OC users within this analytical cohort, and unfortunately within the EPIC cohort, information of dose or type of OC used, date of last use and information on changes to OC use after baseline are not available. Similarly, the estrogens and progestins in oral contraceptives differ in type and concentration [37] and this could be hiding a risk association.

Major strengths of this study are its prospective design and large number of incident cases with receptor information. The large case numbers allowed an in-depth analysis of reproductive-related relative risks, describing risk associations among women of predominantly premenopausal and postmenopausal age. The large case numbers also enabled us to examine subgroup effects such as age at first birth, age at diagnosis, BMI and breastfeeding. To our knowledge, this analysis uses the largest number of incident cases of ER-PR- malignancies, although future prospective studies with a greater number of ER-PR- cases are necessary to characterize the associations, which are of substantially smaller magnitude when compared to their HR-positive counterparts**.** Our study does have its limitations. The determination of ER and PR status in breast tumors has become a standard part of breast cancer diagnosis and is used to predict endocrine therapy response [4]. While a number of studies have shown that the classification of the ER and PR in tumors is relatively robust [38,39], the accuracy of classifying an ER or PR-negative tumor remains controversial [40,41]. In the analysis that compared women with a complete absence of ER and PR expression to women with any indication of an ER and PR positive expression, ER-PR- risk estimates remained unchanged. Furthermore, proportions of ER and PR–negative tumors in the EPIC cohort are in line with previous reports [13,42]. In addition, the inclusion of PR provides an indication of a functional estrogen pathway [2] and thus a joint ER-PR- may be more reflective of a true ER-negative tumor. At the time of this study, additional information on HER2 expression to determine breast cancer subtypes into more detailed molecular sub-classifications could not be completed because of insufficient information on HER2. However, as the routine assessment of HER2 is relatively more recent than ER and PR assessment, future cohort analyses will be able to include HER2.

## Conclusions

In conclusion, our study provides evidence that later age at first full-term childbirth is associated with an increased risk of ER+PR+ tumors but not with ER-PR- tumors. Moreover, age at menarche and time between menarche and first full-term childbirth may be associated with the etiology of both HR-negative and HR-positive malignancies, although associations were only borderline significant for HR-negative tumors. Further studies with more incident cases of ER-PR- tumors are needed to provide more precise risk estimation for reproductive factors with HR-negative tumors.

## Abbreviations

EPIC: European Prospective Investigation into Cancer and Nutrition; ER: Estrogen receptor; PR: Progesterone receptor; HR-positive: Hormone receptor-positive; HR-negative: Hormone receptor-negative; OC: Oral contraception; HR: Hazard ratio; 95% CI: 95% confidence interval; HER2: Human epidermal growth factor-2; BMI: Body mass index.

## Competing interests

The authors declare they have no competing interests.

## Authors' contributions

RR, AL, and RK contributed to the conception of the current analysis and all authors were involved in the design and acquisition of data from the EPIC cohort. RR, AL and RK contributed to the analysis and all authors contributed to the interpretation of the data. RR, KT, AL and RK drafted the manuscript and all authors revised the final draft critically for important critical content. All authors have given final approval of the version to be published.

## Pre-publication history

The pre-publication history for this paper can be accessed here:

http://www.biomedcentral.com/1471-2407/13/584/prepub

## Supplementary Material

Additional file 1: Table S1 Reproductive factors and risk of ER-positive vs. ER-negative and PR-positive vs. PR-negative breast cancer in all women. Click here for file

Additional file 2: Table S2 Reproductive factors and risk of discordant breast cancer subtypes in all women. Click here for file
