# Supplementary material for: Reproductive factors and risk of hormone receptor positive and negative breast cancer: a cohort study
Source: BMC Cancer. 2013 Dec 9;13:584. doi: 10.1186/1471-2407-13-584 (PMC3866571; doi:10.1186/1471-2407-13-584)
Supplement: Additional file 1: Table S1 — Reproductive factors and risk of ER-positive vs. ER-negative and PR-positive vs. PR-negative breast cancer in all women. [file 1471-2407-13-584-S1.docx]

**Additional file 1: Table S1: Reproductive factors and risk of ER-positive vs. ER-negative and PR-positive vs. PR-negative breast cancer in all women**

|  | **Multivariable adjusted^1^** | | | | | | | | | | | |
| --- | --- | --- | --- | --- | --- | --- | --- | --- | --- | --- | --- | --- |
|  | **ER-positive** | | | **ER-negative** | | | **PR-positive** | | | **PR-negative** | | |
|  | **(n = 5723)** | | | **(n = 1372)** | | | **(n = 3776)** | | | **(n = 2083)** | | |
| **Reproductive factor** | **Cases** | **HR** | **95% CI** | **Cases** | **HR** | **95% CI** | **Cases** | **HR** | **95% CI** | **Cases** | **HR** | **95% CI** |
| **Age at menarche** | | | | | | | | | | | | |
| <13 years | 2108 | 1.00 | Reference | 500 | 1.00 | Reference | 1451 | 1.00 | Reference | 760 | 1.00 | Reference |
| 14 years | 2726 | 0.97 | (0.91-1.02) | 657 | 1.00 | (0.89-1.13) | 1821 | 0.97 | (0.90-1.04) | 1016 | 1.01 | (0.92-1.12) |
| ≥15 years | 795 | 0.82 | (0.76-0.90) | 202 | 0.93 | (0.78-1.10) | 466 | 0.78 | (0.70-0.86) | 284 | 0.88 | (0.76-1.01) |
| P for trend |  |  | <0.001 |  |  | 0.48 |  |  | <0.001 |  |  | 0.17 |
| Subtype heterogeneity^2^ |  |  |  |  |  | 0.25 |  |  |  |  |  | 0.18 |
| **Age at menopause^3^** | | | | | | | | | | | | |
| ≤48 years | 787 | 1.00 | Reference | 190 | 1.00 | Reference | 482 | 1.00 | Reference | 277 | 1.00 | Reference |
| 49-50 years | 675 | 1.12 | (1.01-1.25) | 147 | 1.00 | (0.80-1.25) | 409 | 1.12 | (0.97-1.28) | 242 | 1.08 | (0.90-1.30) |
| 51-54 years | 527 | 1.18 | (1.05-1.33) | 80 | 0.82 | (0.62-1.08) | 290 | 1.07 | (0.92-1.24) | 157 | 1.03 | (0.84-1.27) |
| ≥55 years | 231 | 1.31 | (1.13-1.53) | 46 | 1.12 | (0.79-1.56) | 126 | 1.19 | (0.97-1.46) | 88 | 1.44 | (1.11-1.85) |
| P for trend |  |  | <0.001 |  |  | 0.72 |  |  | 0.13 |  |  | 0.04 |
| Subtype heterogeneity^2^ |  |  |  |  |  | 0.06 |  |  |  |  |  | 0.45 |
| **Ever a full-term childbirth** | | | | | | | | | | | | |
| No | 759 | 1.00 | Reference | 157 | 1.00 | Reference | 463 | 1.00 | Reference | 260 | 1.00 | Reference |
| Yes | 4785 | 0.84 | (0.78-0.91) | 1163 | 1.00 | (0.84-1.18) | 3176 | 0.88 | (0.80-0.98) | 1739 | 0.87 | (0.76-0.99) |
| P for significance |  |  | <0.001 |  |  | 0.96 |  |  | 0.02 |  |  | 0.04 |
| Subtype heterogeneity^2^ |  |  |  |  |  | 0.65 |  |  |  |  |  | 0.48 |
| **Number of full-term childbirths^4^** | | | | | | | | | | | | |
| 1 child | 966 | 1.00 | Reference | 219 | 1.00 | Reference | 653 | 1.00 | Reference | 350 | 1.00 | Reference |
| 2 children | 2382 | 0.90 | (0.84-0.97) | 598 | 1.02 | (0.87-1.20) | 1593 | 0.92 | (0.84-1.01) | 867 | 0.92 | (0.81-1.04) |
| >3 children | 1385 | 0.78 | (0.71-0.84) | 338 | 0.93 | (0.78-1.10) | 884 | 0.76 | (0.69-0.85) | 508 | 0.82 | (0.71-0.94) |
| P for trend |  |  | <0.001 |  |  | 0.30 |  |  | <0.001 |  |  | <0.001 |
| Subtype heterogeneity^2^ |  |  |  |  |  | 0.09 |  |  |  |  |  | 0.49 |
| **Age at first full-term childbirth^4^** | | | | | | | | | | | | |
| >19 years | 594 | 1.00 | Reference | 153 | 1.00 | Reference | 376 | 1.00 | Reference | 207 | 1.00 | Reference |
| 20-24 years | 2125 | 1.03 | (0.94-1.13) | 575 | 1.09 | (0.90-1.30) | 1449 | 1.07 | (0.95-1.20) | 815 | 1.05 | (0.90-1.23) |
| 25-29 years | 1496 | 1.23 | (1.12-1.36) | 336 | 1.09 | (0.89-1.34) | 972 | 1.22 | (1.08-1.38) | 526 | 1.16 | (0.98-1.37) |
| 30-34 years | 429 | 1.35 | (1.18-1.54) | 90 | 1.10 | (0.84-1.44) | 296 | 1.39 | (1.19-1.64) | 150 | 1.27 | (1.02-1.58) |
| ≤35 years | 133 | 1.46 | (1.20-1.77) | 20 | 0.89 | (0.56-1.43) | 87 | 1.46 | (1.15-1.86) | 47 | 1.39 | (1.01-1.93) |
| P for trend |  |  | <0.001 |  |  | 0.80 |  |  | <0.001 |  |  | 0.002 |
| Subtype heterogeneity^2^ |  |  |  |  |  | 0.01 |  |  |  |  |  | 0.49 |
| **Time between menarche and first full-term childbirth^4^** | | | | | | | | | | | | |
| <10 years | 1368 | 1.00 | Reference | 366 | 1.00 | Reference | 882 | 1.00 | Reference | 512 | 1.00 | Reference |
| ≥10 years | 3344 | 1.20 | (1.12-1.28) | 801 | 1.09 | (0.95-1.24) | 2273 | 1.22 | (1.13-1.33) | 1219 | 1.12 | (1.00-1.25) |
| P for significance |  |  | <0.001 |  |  | 0.21 |  |  | <0.001 |  |  | 0.045 |
| Subtype heterogeneity^2^ |  |  |  |  |  | 0.18 |  |  |  |  |  | 0.22 |
| **Age at last full-term childbirth^4^** | | | | | | | | | | | | |
| <24 years | 725 | 1.00 | Reference | 188 | 1.00 | Reference | 488 | 1.00 | Reference | 264 | 1.00 | Reference |
| 25-29 years | 1697 | 0.98 | (0.90-1.07) | 454 | 1.03 | (0.87-1.23) | 1129 | 0.99 | (0.89-1.10) | 626 | 0.98 | (0.85-1.13) |
| 30-34 years | 1556 | 1.07 | (0.98-1.17) | 354 | 0.96 | (0.80-1.15) | 1038 | 1.07 | (0.96-1.20) | 545 | 1.00 | (0.86-1.17) |
| >35 years | 801 | 1.16 | (1.04-1.29) | 179 | 1.04 | (0.84-1.29) | 525 | 1.14 | (1.00-1.29) | 312 | 1.20 | (1.01-1.42) |
| P for trend |  |  | <0.001 |  |  | 0.94 |  |  | 0.01 |  |  | 0.03 |
| Subtype heterogeneity^2^ |  |  |  |  |  | 0.10 |  |  |  |  |  | 0.78 |
| **Time since last full-term childbirth^4^** | | | | | | | | | | | | |
| ≤20 years | 1496 | 1.00 | Reference | 409 | 1.00 | Reference | 1130 | 1.00 | Reference | 589 | 1.00 | Reference |
| >20 years | 3283 | 0.84 | (0.77-0.91) | 766 | 1.00 | (0.84-1.18) | 2050 | 0.86 | (0.78-0.95) | 1158 | 0.85 | (0.74-0.98) |
| P for significance |  |  | <0.001 |  |  | 0.96 |  |  | 0.004 |  |  | 0.02 |
| Subtype heterogeneity^2^ |  |  |  |  |  | 0.08 |  |  |  |  |  | 0.85 |
| **Ever breast-fed^4^** | | | | | | | | | | | | |
| No | 828 | 1.00 | Reference | 199 | 1.00 | Reference | 572 | 1.00 | Reference | 324 | 1.00 | Reference |
| Yes | 3741 | 0.97 | (0.90-1.05) | 901 | 1.02 | (0.87-1.20) | 2454 | 0.99 | (0.90-1.09) | 1322 | 0.96 | (0.85-1.09) |
| P for significance |  |  | 0.45 |  |  | 0.81 |  |  | 0.84 |  |  | 0.57 |
| Subtype heterogeneity^2^ |  |  |  |  |  | 0.58 |  |  |  |  |  | 0.74 |
| **Total cumulative duration of breastfeeding^4,5^** | | | | | | | | | | | | |
| <1 month | 419 | 1.00 | Reference | 105 | 1.00 | Reference | 264 | 1.00 | Reference | 144 | 1.00 | Reference |
| 1-3 months | 940 | 1.01 | (0.90-1.14) | 212 | 0.88 | (0.69-1.11) | 640 | 1.04 | (0.90-1.20) | 328 | 0.98 | (0.81-1.20) |
| 13-17 months | 300 | 0.89 | (0.77-1.04) | 83 | 1.06 | (0.78-1.42) | 190 | 0.91 | (0.75-1.11) | 128 | 1.13 | (0.88-1.45) |
| 4-6 months | 777 | 0.98 | (0.87-1.11) | 188 | 0.93 | (0.73-1.19) | 487 | 0.97 | (0.83-1.13) | 293 | 1.05 | (0.86-1.28) |
| 7-12 months | 783 | 0.91 | (0.81-1.03) | 192 | 0.92 | (0.72-1.18) | 518 | 0.98 | (0.84-1.14) | 262 | 0.90 | (0.73-1.11) |
| ≥18 months | 454 | 1.01 | (0.88-1.17) | 110 | 1.10 | (0.83-1.47) | 307 | 1.12 | (0.94-1.34) | 143 | 0.96 | (0.75-1.23) |
| P for trend |  |  | 0.25 |  |  | 0.21 |  |  | 0.80 |  |  | 0.86 |
| Subtype heterogeneity^2^ |  |  |  |  |  | 0.10 |  |  |  |  |  | 0.79 |
| **Ever had an abortion^6^** | | | | | | | | | | | | |
| No | 2590 | 1.00 | Reference | 605 | 1.00 | Reference | 1647 | 1.00 | Reference | 894 | 1.00 | Reference |
| Yes | 1675 | 0.99 | (0.93-1.05) | 424 | 1.05 | (0.93-1.19) | 1086 | 0.99 | (0.92-1.07) | 623 | 1.04 | (0.94-1.15) |
| P for significance |  |  | 0.68 |  |  | 0.44 |  |  | 0.83 |  |  | 0.49 |
| Subtype heterogeneity |  |  |  |  |  | 0.38 |  |  |  |  |  | 0.52 |
| **OC use at recruitment** | | | | | | | | | | | | |
| Never OC user | 2405 | 1.00 | Reference | 506 | 1.00 | Reference | 1553 | 1.00 | Reference | 860 | 1.00 | Reference |
| Past OC user | 2966 | 0.98 | (0.93-1.04) | 762 | 1.12 | (0.99-1.27) | 1953 | 0.97 | (0.91-1.05) | 1089 | 1.02 | (0.92-1.12) |
| Current OC user | 165 | 1.29 | (1.09-1.53) | 47 | 1.20 | (0.86-1.67) | 117 | 1.19 | (0.97-1.46) | 62 | 1.19 | (0.90-1.58) |
| P for trend |  |  | 0.47 |  |  | 0.06 |  |  | 0.86 |  |  | 0.46 |
| Subtype heterogeneity^7^ |  |  |  |  |  | 0.11 |  |  |  |  |  | 0.80 |
| **Age started OC^8^** | | | | | | | | | | | | |
| ≤24 years | 1372 | 1.00 | Reference | 414 | 1.00 | Reference | 961 | 1.00 | Reference | 515 | 1.00 | Reference |
| 25-29 years | 584 | 0.99 | (0.89-1.11) | 129 | 0.82 | (0.66-1.02) | 355 | 0.86 | (0.76-0.99) | 216 | 1.02 | (0.86-1.22) |
| 30-34 years | 505 | 0.99 | (0.88-1.12) | 129 | 0.94 | (0.74-1.20) | 325 | 0.94 | (0.81-1.09) | 170 | 0.92 | (0.75-1.14) |
| ≥35 years | 450 | 1.20 | (1.04-1.38) | 97 | 0.96 | (0.72-1.27) | 280 | 1.09 | (0.92-1.29) | 178 | 1.25 | (1.00-1.56) |
| P for trend |  |  | 0.05 |  |  | 0.68 |  |  | 0.61 |  |  | 0.20 |
| Subtype heterogeneity^2^ |  |  |  |  |  | 0.21 |  |  |  |  |  | 0.47 |
| **Duration of OC use^8^** | | | | | | | | | | | | |
| 1 year or less | 650 | 1.00 | Reference | 153 | 1.00 | Reference | 418 | 1.00 | Reference | 245 | 1.00 | Reference |
| 2-4 years | 717 | 0.97 | (0.87-1.08) | 167 | 0.92 | (0.74-1.15) | 476 | 1.03 | (0.90-1.17) | 248 | 0.89 | (0.74-1.06) |
| 5-9 years | 660 | 0.97 | (0.87-1.08) | 169 | 1.02 | (0.81-1.27) | 454 | 1.08 | (0.94-1.23) | 235 | 0.91 | (0.76-1.09) |
| >10 years | 857 | 0.96 | (0.86-1.06) | 266 | 1.20 | (0.97-1.48) | 564 | 1.06 | (0.93-1.21) | 346 | 1.03 | (0.87-1.22) |
| P for trend |  |  | 0.43 |  |  | 0.03 |  |  | 0.31 |  |  | 0.57 |
| Subtype heterogeneity^2^ |  |  |  |  |  | 0.02 |  |  |  |  |  | 0.89 |
| 1 Stratified by age at recruitment and center and further adjusted for BMI, height, menopausal status at enrolment, HRT use, physical activity, smoking status, alcohol consumption and attained level of education; 2 heterogeneity between ER+PR+ and ER-PR- tumors was assessed on the trend score using the data augmentation method as described by Lunn and McNeil; 3 in postmenopausal women only; 4 in parous women only; 5 in women who breast-fed only; 6 in both spontaneous and induced abortions; 7 heterogeneity between ER+PR+ and ER-PR- tumors was assessed on the unordered categorical variable of never, past and current OC use using the data augmentation method as described by Lunn and McNeil; 8 in women who ever used OC. | | | | | | | | | | | | |
