# Supplementary material for: Reproductive factors and risk of hormone receptor positive and negative breast cancer: a cohort study
Source: BMC Cancer. 2013 Dec 9;13:584. doi: 10.1186/1471-2407-13-584 (PMC3866571; doi:10.1186/1471-2407-13-584)
Supplement: Additional file 2: Table S2 — Reproductive factors and risk of discordant breast cancer subtypes in all women. [file 1471-2407-13-584-S2.docx]

**Additional file 2: Table S2: Reproductive factors and risk of discordant breast cancer subtypes in all women**

|  | **Multivariable adjusted^1^** | | | | | | | | |
| --- | --- | --- | --- | --- | --- | --- | --- | --- | --- |
|  | **ER+PR-** | | | **ER-PR+** | | | **ER or PR missing** | | |
|  | **(n = 1078)** | | | **(n = 200)** | | | **(n = 3613)** | | |
| **Reproductive factor** | **Cases** | **HR** | **95% CI** | **Cases** | **HR** | **95% CI** | **Cases** | **HR** | **95% CI** |
| **Age at menarche** | | | | | | | | | |
| ≤13 years | 384 | 1.00 | Reference | 75 | 1.00 | Reference | 1,247 | 1.00 | Reference |
| 14 years | 529 | 1.04 | (0.91-1.18) | 99 | 1.09 | (0.80-1.48) | 1,693 | 0.98 | (0.91-1.05) |
| ≥15 years | 148 | 0.90 | (0.74-1.10) | 26 | 1.04 | (0.65-1.64) | 599 | 0.89 | (0.80-0.98) |
| P for trend |  |  | 0.51 |  |  | 0.74 |  |  | 0.03 |
| **Age at menopause^2^** | | | | | | | | | |
| ≤48 years | 140 | 1.00 | Reference | 16 | 1.00 | Reference | 575 | 1.00 | Reference |
| 49-50 years | 125 | 1.06 | (0.82-1.36) | 11 | 1.02 | (0.44-2.33) | 458 | 1.09 | (0.96-1.24) |
| 51-54 years | 93 | 1.14 | (0.87-1.50) | 8 | 1.05 | (0.41-2.64) | 386 | 1.26 | (1.10-1.44) |
| ≥55 years | 56 | 1.85 | (1.33-2.58) | 5 | 2.10 | (0.69-6.36) | 152 | 1.27 | (1.06-1.53) |
| P for trend |  |  | 0.00 |  |  | 0.36 |  |  | <0.001 |
| **Ever a full-term childbirth** | | | | | | | | | |
| No | 145 | 1.00 | Reference | 16 | 1.00 | Reference | 556 | 1.00 | Reference |
| Yes | 889 | 0.78 | (0.65-0.93) | 174 | 1.36 | (0.81-2.29) | 3,000 | 0.85 | (0.77-0.93) |
| P for significance |  |  | 0.01 |  |  | 0.25 |  |  | <0.001 |
| **Number of full-term childbirths^3^** | | | | | | | | | |
| 1 child | 190 | 1.00 | Reference | 40 | 1.00 | Reference | 542 | 1.00 | Reference |
| 2 children | 432 | 0.84 | (0.70-0.99) | 91 | 0.91 | (0.62-1.33) | 1,455 | 0.97 | (0.88-1.07) |
| ≥3 children | 260 | 0.74 | (0.61-0.90) | 42 | 0.80 | (0.51-1.25) | 894 | 0.87 | (0.78-0.97) |
| P for trend |  |  | 0.00 |  |  | 0.32 |  |  | 0.01 |
| **Age at first full-term childbirth ^3^** | | | | | | | | | |
| ≤19 years | 104 | 1.00 | Reference | 18 | 1.00 | Reference | 421 | 1.00 | Reference |
| 20-24 years | 381 | 0.91 | (0.73-1.13) | 79 | 1.00 | (0.59-1.68) | 1,267 | 1.00 | (0.89-1.12) |
| 25-29 years | 282 | 1.12 | (0.89-1.43) | 57 | 1.16 | (0.67-2.02) | 972 | 1.24 | (1.10-1.40) |
| 30-34 years | 88 | 1.36 | (1.01-1.83) | 19 | 1.38 | (0.70-2.70) | 261 | 1.21 | (1.03-1.42) |
| ≥35 years | 32 | 1.70 | (1.13-2.55) | 4 | 1.19 | (0.40-3.59) | 71 | 1.13 | (0.87-1.46) |
| P for trend |  |  | <0.001 |  |  | 0.23 |  |  | <0.001 |
| **Time between menarche and first full-term childbirth ^3^** | | | | | | | | | |
| ≤10 years | 254 | 1.00 | Reference | 43 | 1.00 | Reference | 932 | 1.00 | Reference |
| >10 years | 621 | 1.08 | (0.93-1.26) | 134 | 1.24 | (0.87-1.77) | 2,004 | 1.14 | (1.05-1.24) |
| P for significance |  |  | 0.32 |  |  | 0.24 |  |  | 0.00 |
| **Age at last full-term childbirth ^3^** | | | | | | | | | |
| ≤24 years | 126 | 1.00 | Reference | 25 | 1.00 | Reference | 481 | 1.00 | Reference |
| 25-29 years | 303 | 0.95 | (0.77-1.17) | 70 | 1.13 | (0.71-1.79) | 1,090 | 1.04 | (0.93-1.16) |
| 30-34 years | 280 | 1.02 | (0.82-1.27) | 48 | 0.89 | (0.54-1.45) | 980 | 1.12 | (1.00-1.26) |
| ≥35 years | 179 | 1.32 | (1.04-1.68) | 34 | 1.39 | (0.81-2.37) | 443 | 1.06 | (0.92-1.21) |
| P for trend |  |  | 0.01 |  |  | 0.56 |  |  | 0.15 |
| **Time since last full-term childbirth ^3^** | | | | | | | | | |
| ≤20 years | 286 | 1.00 | Reference | 85 | 1.00 | Reference | 811 | 1.00 | Reference |
| >20 years | 602 | 0.76 | (0.64-0.92) | 92 | 0.92 | (0.61-1.37) | 2,183 | 0.91 | (0.81-1.03) |
| P for trend |  |  | 0.01 |  |  | 0.67 |  |  | 0.14 |
| **Ever breast-fed^3^** | | | | | | | | | |
| No | 171 | 1.00 | Reference | 34 | 1.00 | Reference | 431 | 1.00 | Reference |
| Yes | 674 | 0.95 | (0.80-1.13) | 129 | 1.02 | (0.69-1.50) | 2,378 | 0.94 | (0.84-1.04) |
| P for significance |  |  | 0.59 |  |  | 0.94 |  |  | 0.20 |
| **Total cumulative breastfeeding duration^3,4^** | | | | | | | | | |
| ≤1 month | 72 | 1.00 | Reference | 15 | 1.00 | Reference | 301 | 1.00 | Reference |
| 1-3 months | 172 | 1.04 | (0.79-1.37) | 35 | 0.96 | (0.52-1.77) | 550 | 0.91 | (0.79-1.04) |
| 4-6 months | 154 | 1.08 | (0.81-1.44) | 26 | 0.89 | (0.47-1.70) | 453 | 0.91 | (0.78-1.06) |
| 7-12 months | 129 | 0.86 | (0.64-1.16) | 29 | 1.07 | (0.57-2.04) | 503 | 0.88 | (0.76-1.02) |
| 13-17 months | 64 | 1.09 | (0.77-1.54) | 7 | 0.72 | (0.29-1.82) | 221 | 0.92 | (0.77-1.10) |
| ≥18 months | 66 | 0.83 | (0.58-1.19) | 13 | 1.33 | (0.59-2.99) | 326 | 0.93 | (0.79-1.10) |
| P for trend |  |  | 0.21 |  |  | 0.67 |  |  | 0.56 |
| **Ever an abortion^5^** | | | | | | | | | |
| No | 454 | 1.00 | Reference | 90 | 1.00 | Reference | 1,683 | 1.00 | Reference |
| Yes | 328 | 1.08 | (0.94-1.25) | 67 | 1.10 | (0.80-1.51) | 1,072 | 1.00 | (0.92-1.08) |
| P for significance |  |  | 0.29 |  |  | 0.57 |  |  | 0.94 |
| **OC use at recruitment** | | | | | | | | | |
| Never OC user | 478 | 1.00 | Reference | 69 | 1.00 | Reference | 1,457 | 1.00 | Reference |
| Past OC user | 537 | 0.98 | (0.85-1.12) | 112 | 1.12 | (0.81-1.56) | 1,950 | 1.05 | (0.97-1.13) |
| Current OC user | 29 | 1.38 | (0.93-2.06) | 9 | 1.33 | (0.63-2.81) | 137 | 1.23 | (1.01-1.50) |
| **Age start OC use^6^** | | | | | | | | | |
| ≤24 years | 221 | 1.00 | Reference | 62 | 1.00 | Reference | 1,023 | 1.00 | Reference |
| 25-29 years | 121 | 1.20 | (0.94-1.54) | 19 | 0.73 | (0.42-1.26) | 394 | 1.11 | (0.97-1.26) |
| 30-34 years | 79 | 0.93 | (0.69-1.25) | 21 | 1.03 | (0.58-1.83) | 353 | 1.08 | (0.93-1.25) |
| ≥35 years | 105 | 1.58 | (1.17-2.14) | 15 | 1.04 | (0.54-2.04) | 231 | 0.97 | (0.81-1.18) |
| P for trend |  |  | 0.03 | . | . | 0.88 |  |  | 0.86 |
| **OC use duration^6^** | | | | | | | | | |
| 1 year or less | 130 | 1.00 | Reference | 22 | 1.00 | Reference | 371 | 1.00 | Reference |
| 2-4 years | 131 | 0.91 | (0.71-1.16) | 25 | 1.05 | (0.59-1.86) | 481 | 1.02 | (0.89-1.17) |
| 5-9 years | 113 | 0.86 | (0.66-1.11) | 28 | 1.35 | (0.76-2.40) | 443 | 0.95 | (0.82-1.09) |
| ≥10 years | 157 | 0.95 | (0.75-1.22) | 42 | 1.81 | (1.05-3.12) | 663 | 0.93 | (0.81-1.06) |
| P for trend |  |  | 0.69 |  |  | 0.02 |  |  | 0.14 |
| ^1^ Stratified by age at recruitment and center and further adjusted for BMI, height, menopausal status at enrolment, HRT use, physical activity, smoking status, alcohol consumption and attained level of education; ^2^ in postmenopausal women only; ^3^ in parous women only; ^4^ in women who breast-fed only; ^5^ in both spontaneous and induced abortions; ^6^ in women who used OC. | | | | | | | | | |
